# Enhancement of immune response against *Bordetella* spp. by disrupting immunomodulation

**DOI:** 10.1038/s41598-019-56652-z

**Published:** 2019-12-30

**Authors:** Monica C. Gestal, Laura K. Howard, Kalyan Dewan, Hannah M. Johnson, Mariette Barbier, Clare Bryant, Illiassou Hamidou Soumana, Israel Rivera, Bodo Linz, Uriel Blas-Machado, Eric T. Harvill

**Affiliations:** 1 Department of Infectious Diseases, College of Veterinary Medicine, University of Georgia, Athens, Georgia United States of America; 2 Department of Microbiology, Immunology, and Cell Biology, West Virginia University, Morgantown, WV United States of America; 3 Vaccine Development Center at West Virginia University Health Sciences Center, Morgantown, West Virginia United States of America; 4 Department of Veterinary Medicine, University of Cambridge, Cambridge, CB3 0ES United Kingdom; 5 Department of Pathology, Athens Veterinary Diagnostic Laboratory, University of Georgia, Athens, Georgia United States of America

**Keywords:** Infection, Bacterial infection, Bacterial host response, Bacterial immune evasion, Pathogens

## Abstract

Well-adapted pathogens must evade clearance by the host immune system and the study of how they do this has revealed myriad complex strategies and mechanisms. Classical bordetellae are very closely related subspecies that are known to modulate adaptive immunity in a variety of ways, permitting them to either persist for life or repeatedly infect the same host. Exploring the hypothesis that exposure to immune cells would cause bordetellae to induce expression of important immunomodulatory mechanisms, we identified a putative regulator of an immunomodulatory pathway. The deletion of *btrS* in *B. bronchiseptica* did not affect colonization or initial growth in the respiratory tract of mice, its natural host, but did increase activation of the inflammasome pathway, and recruitment of inflammatory cells. The mutant lacking *btrS* recruited many more B and T cells into the lungs, where they rapidly formed highly organized and distinctive Bronchial Associated Lymphoid Tissue (BALT) not induced by any wild type *Bordetella* species, and a much more rapid and strong antibody response than observed with any of these species. Immunity induced by the mutant was measurably more robust in all respiratory organs, providing completely sterilizing immunity that protected against challenge infections for many months. Moreover, the mutant induced sterilizing immunity against infection with other classical bordetellae, including *B. pertussis* and *B. parapertussis*, something the current vaccines do not provide. These findings reveal profound immunomodulation by bordetellae and demonstrate that by disrupting it much more robust protective immunity can be generated, providing a pathway to greatly improve vaccines and preventive treatments against these important pathogens.

## Introduction

Pathogens are under natural selection for the ability to colonize and persist in their host, and have evolved complex mechanisms of immune modulation that to date we only poorly recognize, appreciate or understand. Natural host experimental systems have allowed some of these mechanisms to be studied in detail in classical bordetellae, which include *Bordetella* species that are very successful respiratory pathogens of humans and other animals including mice, in which there are sophisticated molecular tools for experimental manipulation of host immunity. In this system these tools have been used to define the contribution of each immune function to the control and clearance of infection. In the context of such studies, we and others have observed that various *Bordetella* species manipulate immunity in a variety of ways that would be undetectable in other experimental systems^1–25^. This immunomodulation allows each *Bordetella* species to achieve remarkable success by either persisting for life with limited pathology (*B. bronchiseptica*) or causing acute coughing disease that facilitates extraordinarily rapid transmission to new hosts (*B. pertussis*)^26,27^. Despite their divergent ecological strategies and niches, these species share a large set of genes whose products mediate interactions with the host, modulating host immunity in various ways to optimize their success. The importance of their many immunomodulatory mechanisms is only beginning to be appreciated^10^.

We recently hypothesized that an aspect of the success of several pathogens including *Bordetella* species involve their *in vivo* differential regulation of an array of host-manipulating factors^28^, each of which are poorly understood. In exploring host signals that might alert the bacteria of both anti-bacterial challenges and immune-modulating opportunities, we and others examined how bordetellae respond to blood components, a signal of inflammation and/or access to deeper body tissues^28–31^. Blood contains a constellation of antimicrobials, immunological signals and immune cells. We have previously reported that *Bordetella* spp. are able to respond to blood and serum by differentially regulating several sets of genes^28^, including a SigE-type sigma factor annotated as *brpL*^32,33^ that was up-regulated 6-fold in blood and 3-fold in serum. This gene, located in the same operon as Type 3 Secretion System (T3SS) regulators such as *btrV/btrW*^34,35^, was previously shown to work in conjunction with BtrA (anti-sigma factor) to regulate the expression of the T3SS and was therefore renamed *btrS*^35,36^. However, our transcriptomic analysis revealed that there is a broader set of genes whose expression is potentially affected by BtrS. These include autotransporters and ABC transport systems, regulatory proteins, and proteins involved in various metabolic functions. Interestingly, genes required for pertussis toxin production (*ptxA* to *-E*) and secretion (*ptlA* to *-L*), previously thought to be quiescent in *B. bronchiseptica*, were also found regulated by BtrS. Altogether, these data suggest that BtrS regulates the T3SS as well as a set of other more poorly characterized genes implicated in immunomodulation.

Here we describe a STRING analysis that associated *btrS*^28,36^ with various genes including some known immunomodulatory factors, implicating BtrS as a potential regulator of immunomodulatory functions. Deleting *btrS* altered *in vitro* expression of T3SS genes as well as genes encoding motility, outer membrane proteins, transporters, and other genes with undefined functions, many of which have the potential to affect the host immune response in a variety of ways. Deleting *btrS* did not affect the ability of the bacterium to colonize and grow in the respiratory tract, but did profoundly prevent the inhibition of inflammasome activation, and resulted in greater inflammation and local immune responses. The mutant was not defective in colonizing or persisting in mice lacking B and T cells, indicating that *btrS* is not required for early steps of the infection process, but is primarily involved in immunomodulation that inhibits effective protective anamnestic immunity. The mutant induced the rapid recruitment of larger numbers of B and T cells that efficiently and rapidly assembled into pronounced Bronchus-Associated Lymphoid Tissue (BALT). The resulting rapid immune response produced much higher antibody titers and allowed mice to completely clear infections with the deletion mutant, while the wild type strain persisted indefinitely. Furthermore, mice convalescent from infection with this mutant were protected from re-infection with all three wild-type classical *Bordetella* species, being much better protected against both *B. pertussis* and *B. parapertussis* than mice given the current vaccine.

## Results

### *btrS* contributes to immunomodulation

**Figure 1:**
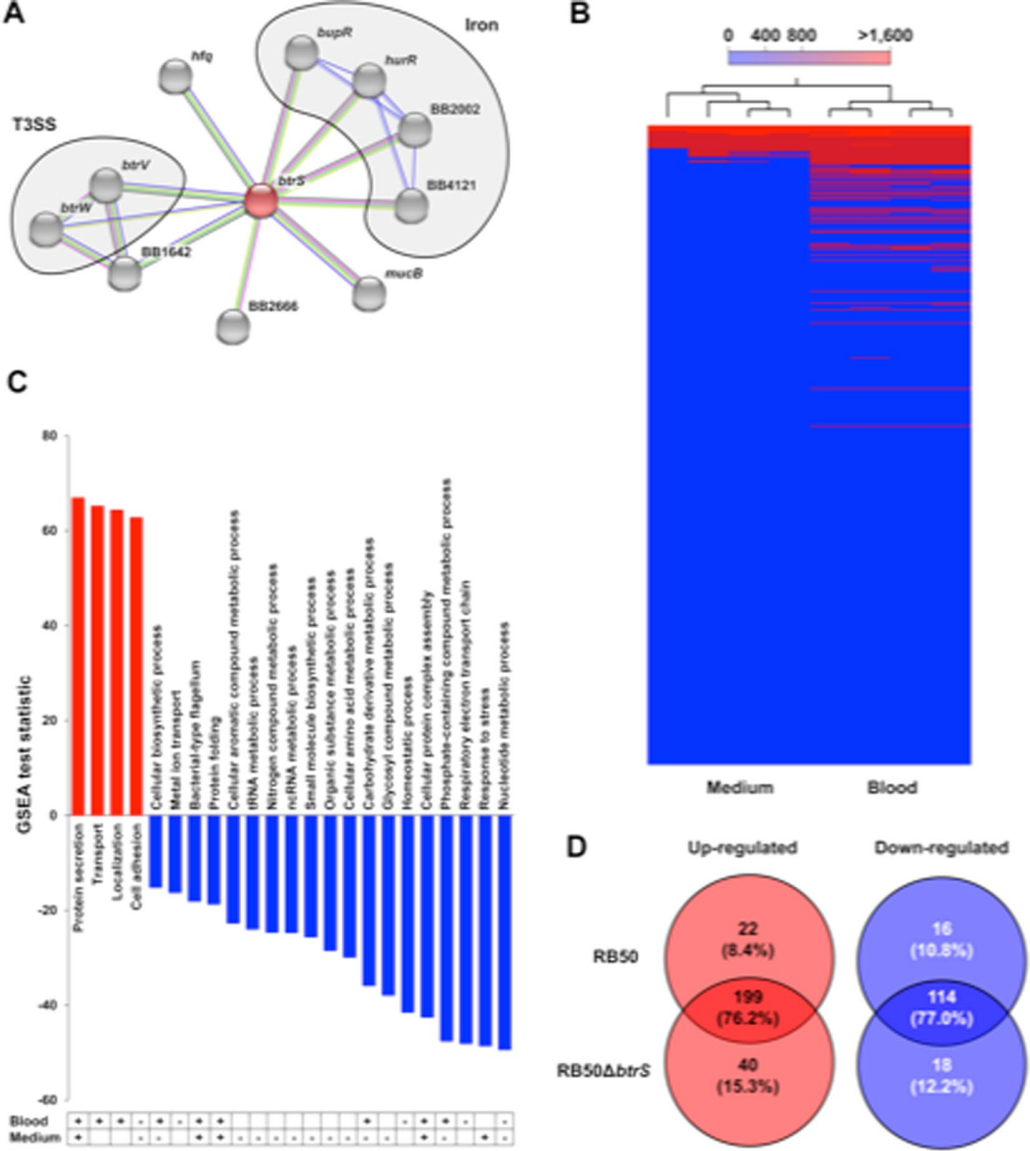
Transcriptional regulation role of *btrS* in medium and serum. STRING analysis of the *btrS* sigma factor. (**A**) Connections are coded as follow: pink and blue show the known connections, where pink shows gene fusions and blue gene co-occurrence; green indicates gene neighborhood, yellow shows textmining, black shows co-expression, and finally purple shows protein homology. Heat map of the genes differentially regulated in response to blood vs. medium in RB50 measured by RNA sequencing (**B**), blue is associated with low expression. Selected statistically significantly up- (red) or down-regulated (blue) Gene Ontology (GO) terms of the genes shown in B. (**C**) The table below reflects whether these terms are positively (+) or negatively (−) regulated by BtrS in blood or medium +/−. Overlap between the genes significantly up-regulated (red) and down-regulated (blue) in RB50Δ*btrS* compared to RB50 in blood (**D**).

We recently showed that bordetellae can detect signals in blood/serum and respond by altering expression of various genes, upregulating expression of the gene *btrS*^35,36^ (*brpL*^32,33^), among others^28^. BtrS has been shown to be a sigma factor that interacts with at least one anti-sigma factor to regulate the T3SS^34–38^. The particular ability of sigma/anti-sigma factor combinations to integrate input from multiple and branched signaling pathways led us to consider BtrS as a potentially pivotal regulator of the complex response to dynamic signals from diverse and rapidly changing host environments as *Bordetella* infection and the immune response it induces develop over time in different locations within the host. To further examine its potential status as an integrator of diverse input signals, we evaluated its association with other known and putative immunomodulators by STRING (Fig. 1A) which mines databases for data on gene neighborhood, gene fusion, co-occurrence, co-expression, protein homology, and text mining. This analysis revealed a network with connections to many other factors, some of which are known to be involved in complex interactions with the host. In addition to T3SS, this analysis indicated that *btrS* is connected with other genes encoding known virulence regulators such as *hfq*^39,40^ and *sigE* (RpoE)^41,42^, as well as iron and heme sensors^30,43,44^, transporters, and other membrane proteins. Roles for some of these factors in immunomodulation and/or pathogenesis of several organisms has been reported^45–59^. We also observed that *btrS* was up-regulated 2.5-fold when *B. bronchiseptica* was internalized in macrophages^60^, revealing responsiveness to interactions with immune cells. Altogether, these data led us to speculate that *btrS* is not only a regulator of the T3SS, but potentially integrates multiple, diverse signals to coordinate the regulation of numerous immunomodulatory factors that must be carefully controlled in a complex, choreographed manner to optimize bacterial success in the challenging environment of the host immune response.

### *btrS* is required for the regulation of multiple virulence and immunomodulatory factors

Previous studies assessed the role of BtrA/S in the regulation of nearby T3SS genes^34–36^; however, the role of *btrS* as a regulator of other genes has not yet been studied. To identify *btrS*-regulated genes, we generated a clean, in-frame deletion mutant (Fig. S1) and performed an RNAseq analysis of wild-type (RB50) and the mutant (RB50Δ*btrS*) after 1 hour incubation in LB media or in blood at 37 °C. Incubation in blood for 1 hour had a profound effect on the transcriptome, altering expression of approximately 7% of the genes (Fig. 1B–D and Supplementary Dataset 1). Genes associated with T3SS products, iron acquisition, as well as protein transport and localization were significantly induced upon blood exposure in the wild-type strain, but not the mutant (Fig. 1C), revealing a role for BtrS in their induction. On the other hand, flagellum biosynthesis and metabolism (nucleic acids, carbohydrates, organic compounds, phosphate-containing compounds, and glycosylated compounds) genes were significantly down-regulated in response to blood in the wild type bacteria, but not the mutant, implicating BtrS in their regulation (Fig. 1D). To test whether these changes in transcript levels translate into differences in phenotype we examined flagellin-dependent motility. The *btrS* null-mutant was significantly more motile than its wild-type parent (Fig. S3B), supporting the RNAseq data and highlighting the differences in flagella expression that might affect interactions with the host, as examined further below. These data indicate that in addition to T3SS, BtrS is involved, directly or indirectly, in the regulation of multiple genes involved in motility, membrane proteins, and toxin production, as well as multiple genes of unknown function.

### *btrS* plays an important role in internalization and survival within RAW macrophages

**Figure 2:**
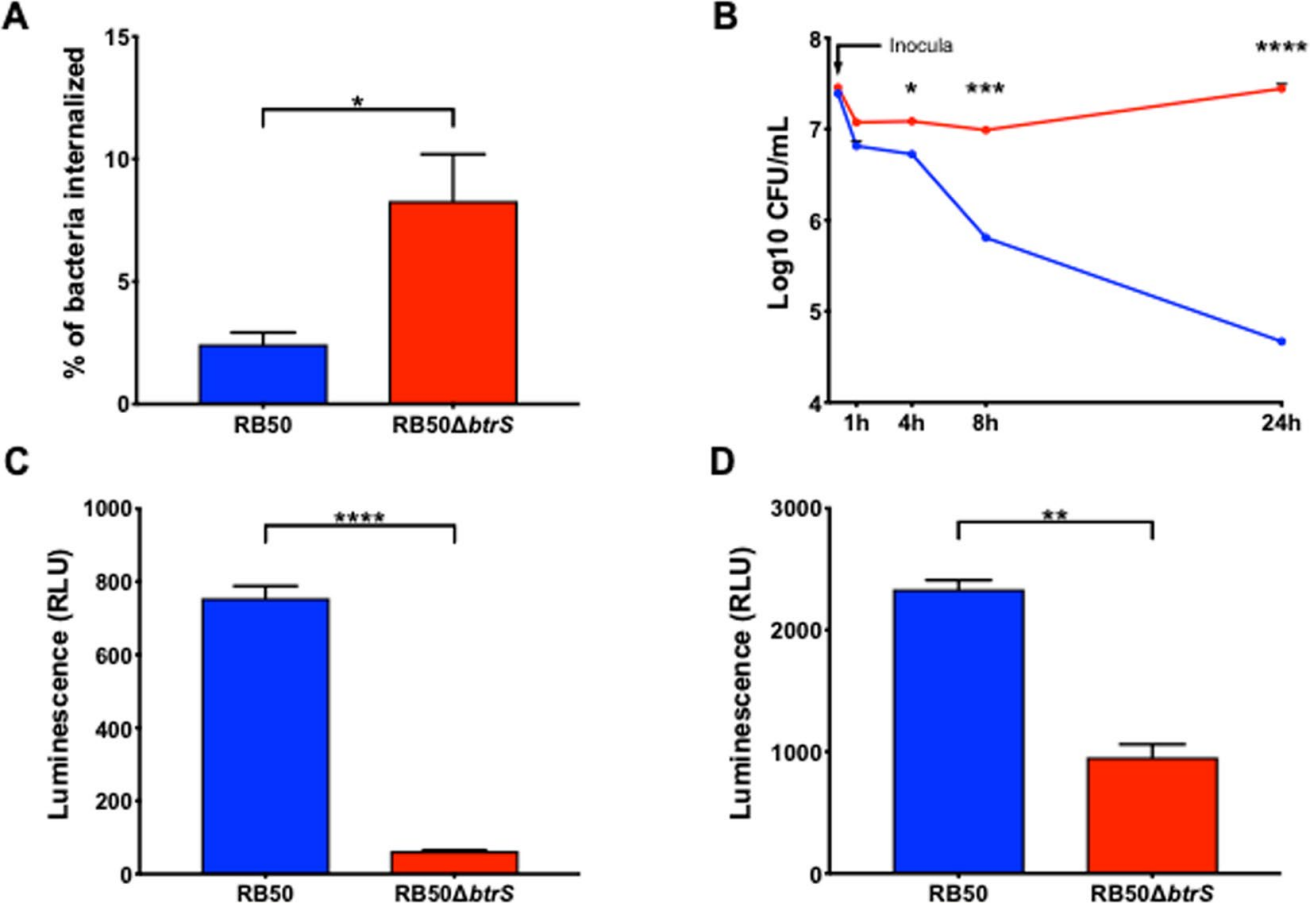
*btrS* mediates intracellular survival in macrophages. Internalization and survival of RB50 (blue) and RB50D*btrS* (red) in RAW 264.7 was measured at 15 minutes (n = 4) (**A**), and up to 24 hours (n = 6) (**B**), respectively. Caspase-1 activity, as measured via luminescence with the Caspase-Glo 1 Inflammasome Assay, induced by RB50 and RB50D*btrS* in THP-1 human-derived macrophages (**C**) and mouse bone marrow-derived macrophages (**D**) within 1 hour (n = 6). Statistical significance was calculated using Two-Way ANOVA. *p < 0.05, **p < 0.005, ***p < 0.0005, and ****p < 0.0001. Error bars indicate SEM.

Based on the analyses above, we hypothesized that the RB50Δ*btrS* mutant will fail to coordinate factors involved in complex interactions with its host. To investigate potential roles in interactions with immune cells, we inoculated RAW 264.7 macrophages with wild type or Δ*btrS* mutant at Multiplicity of Infection (MOI) of 100 (Fig. S2). 15 minutes later, less than 2.5% of the wild type bacteria were internalized, while 8.3% of the Δ*btrS* mutant bacteria were intracellular (Fig. 2A). 24 hours post-inoculation (p.i.), the numbers of wild-type bacteria were reduced by >95%, while the RB50Δ*btrS* mutant not only survived but grew in numbers within macrophages (Fig. 2B). This apparent gain of function, albeit in an artificial *in vitro* assay, was unexpected as we hypothesized that the deletion mutant strain would be defective in survival due to the loss of expression of several virulence factors.

Transmission Electron Microscopy (TEM) revealed that macrophages challenged with the mutant strain in the assay above contained as many as 22 bacteria in the cytoplasm 4 hours later, while those challenged with the wild-type strain contained no more than 8 bacteria within them (Fig. S2B,C). However, the deletion of *btrS* significantly reduced bacterial cytotoxicity against macrophages, with the mutant killing less than 5% of macrophages while the wild-type killed nearly 70% (Fig. S2C), as we and others have previously shown^5,31,61^. Since dead cells do not effectively harbor and protect intracellular bacteria, the decrease in cytotoxicity can partially explain the greater recovery of the mutant within macrophages at 4 hours, but does not explain the noted increase in CFUs recovered in intracellular bacteria over 24 hours. Together, these results reveal that *btrS* contributes significantly to macrophage cytotoxicity, and affects internalization and growth within macrophages, suggesting that the two effects may be potentially related (Fig. S2D). These effects implicate *btrS* in the regulation of interactions of *B. bronchiseptica* with macrophages, which play multiple important roles in generating and shaping the protective immune response to infection.

### Deleting *btrS* alters induction of macrophage immune signaling pathways

To investigate the role of *btrS* in the transcriptional activation of macrophages, we performed an RNAseq analysis of RAW 264.7 macrophages at 15 minutes and 4 hours post-challenge with either wild type or Δ*btrS* mutant. IPA analysis of canonical pathways (Qiagen)^62^ revealed multiple macrophage-signaling pathways that are differentially expressed following challenge with the two strains. While wild-type increased the expression of genes associated with the inflammasome and ephrin pathways, the Δ*btrS* mutant increased expression of pathways involved in macropinocytosis, interferons, innate immunity, and T cell activation/differentiation (Supplementary Dataset 2). Differential induction of these by the mutant indicates that in wild-type *B. bronchiseptica, btrS* mediates the suppression of this important set of immune-related genes.

To more directly examine the effects on inflammation, we challenged macrophages with wild-type or mutant bacteria for 15 minutes and assessed expression of IL-1β (Fig. S2E and Supplementary Dataset 2) and several other genes involved in apoptosis and cell death. All these were strongly up-regulated by wild type bacteria, indicating activation of the inflammasome pathway and an oxidative stress response, as well as positive regulation of TNF-α production (Supplementary Dataset 2). Interestingly, macrophages challenged with the Δ*btrS* mutant increased expression of cytokines and chemokines associated with an increase in immune cell recruitment, NF-|B signaling (pro-inflammatory response), and other factors that positively regulate the activation of an adaptive immune response (T cell activation, leukocyte differentiation, leukocyte activation, and others). The Δ*btrS* mutant also activated genes associated other pathways such as hematopoiesis (Supplementary Dataset 2).

To further investigate the effects of exposure to wild-type or Δ*btrS* mutant bacteria, we performed an RNAseq analysis of macrophages after 4 hours post-inoculation. RB50-challenged macrophages mostly activated genes involved in T cell migration. Macrophages inoculated with RB50Δ*btrS* increased the expression of genes involved in innate immunity such as antigen presentation or positive regulation of the acute inflammatory response, as well as genes involved in adaptive immunity, including lymphocyte proliferation, differentiation, and activation. Together, these assays revealed that the Δ*btrS mutant*, relative to the wild type strain, induces a more profound increase in the expression of genes involved in stimulating various aspects of immunity. Altogether, these results suggest that in the wild-type bacteria, BtrS plays a role in suppressing various aspects of inflammation and immune signaling pathways, potentially modulating the development of effective adaptive immunity.

### *btrS* induces inflammasome activation

The up-regulation of genes related to the inflammasome pathway led us to investigate the role of *btrS* in its activation, a critical aspect of both inflammation and the generation of adaptive responses^63–65^. To do so, we measured caspase-1 levels in the human-derived macrophage-like cell line THP-1 and primary murine bone marrow-derived macrophages. 30 minutes post-inoculation, THP-1 cells challenged with RB50 (Fig. 2C) contained approximately 8-fold more caspase-1 compared with those infected with the deletion mutant. 2 hours post-challenge, murine bone marrow-derived macrophages inoculated with wild type bacteria expressed 2-fold more caspase-1 than those challenged with the mutant (Fig. 2D). These results in both human- and mouse-derived macrophages demonstrate that the inflammasome pathway is substantially induced by RB50 but not by the mutant strain, indicating that BtrS mediates robust activation of the inflammasome pathway.

### *btrS* is required for respiratory tract persistence

**Figure 3:**
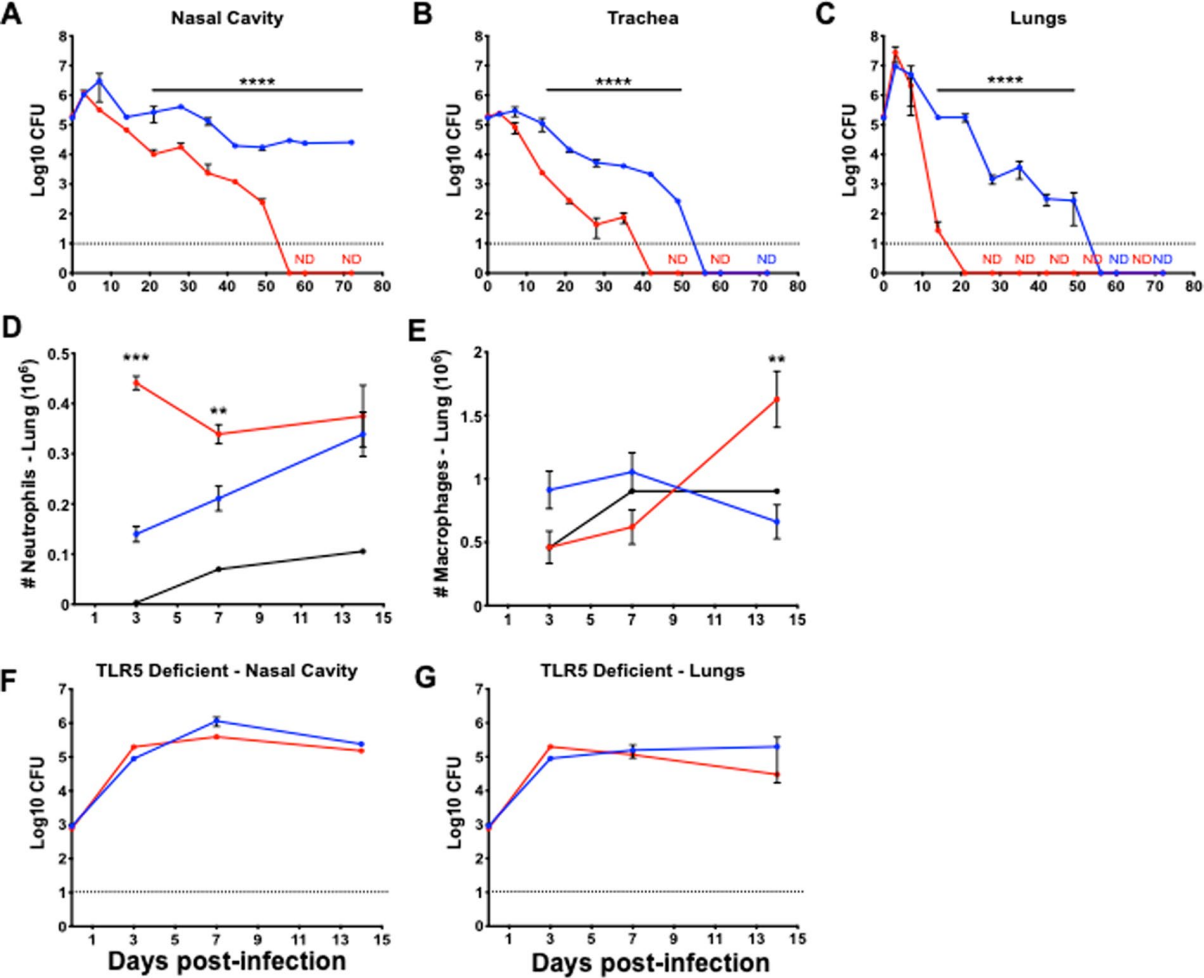
*btrS* promoted persistence in the respiratory tract by suppressing TLR5 mediated clearance. Recovery of *B. bronchiseptica* RB50 (blue) or RB50Δ*btrS* (red) from the respiratory tract of C57Bl/6 J mice, nasal cavity (**A**), trachea (**B**), and lungs (**C**), over time (4–6 mice per experiment, n = 5). Number of neutrophils (**D**) and macrophages (**E**) was evaluated in the lungs (n = 5). Recovery of RB50 or RB50Δ*btrS* bacteria from the nasal cavity (**F**) and lungs (**G**) of TLR5 deficient mice over time (4 mice per group). X-axis indicates days post-inoculation. Statistical significance was calculated using Two-Way ANOVA. *p < 0.05, **p < 0.005, ***p < 0.0005, and ****p < 0.0001. Error bars indicate SEM, ND stands for Not Detected. The dotted line indicates the limit of detection.

The effect of *btrS* deletion on various *in vitro* measures of aspects of interactions with host cells paints a complex picture difficult to extrapolate to understanding its true *in vivo* role(s). The *B. bronchiseptica* natural host infection model in mice allowed us to evaluate this more directly. We therefore followed the course of infection of wild-type and mutant strains in wild type C57BL/6J mice (Fig. 3). Both strains efficiently colonized and grew in the lower respiratory tract during the first few days of infection, indicating that *btrS* is not required for colonization or growth in the host (Fig. 3B,C). However, after one week, when adaptive immunity begins to be detectable^66,67^, the mutant began to demonstrate profound defects in persistence throughout the respiratory tract. Wild-type bacteria persisted in the lungs up to 56 days, whereas the mutant was nearly cleared by day 14 and was undetectable by day 21 (Fig. 3C). Even more striking was the phenotype of the mutant in the nasal cavity (Fig. 3A). As expected, wild-type *B. bronchiseptica* persisted in the nasal cavity of mice for at least 56 days in this experiment, consistent with observations from many previous experiments demonstrating that this wild-type strain always persists indefinitely in the nares of mice^41,68^. In contrast, the *btrS* mutant was completely cleared from the nasal cavity by day 56. This is the first *B. bronchiseptica* mutant described that efficiently colonizes and grows within mice, but is completely cleared from the upper and lower respiratory tract^23,36,69,70^. To rigorously verify that this profound defect of the Δ*btrS* mutant is not due to any other mutation, we compared wild-type and Δ*btrS* mutant, to the Δ*btrS* complemented with *btrS* contained on the plasmid pBBR1MCS (Δ*btrS::btrS* complemented strain). After 28 days complemented strain persisted in the respiratory tract of mice, confirming that this profound defect in persistence is due to the Δ*btrS* deletion (Fig. S3A). This phenotype is much more profound than that reported for a mutant lacking a functional T3SS, indicating that BtrS mediates persistence via effects involving other genes.

### *btrS* mediates decreases in neutrophil and macrophage recruitment to lungs

The remarkable early clearance of the mutant suggests that *btrS* mediates the modulation of the functions of immune effector cells that can otherwise clear infection. This is consistent with our observation that macrophages infected with the *btrS* mutant have increased expression of chemokines such as PPBP (neutrophil recruitment) and CCL7 (macrophage recruitment) (Supplementary Dataset 2). To evaluate the effect of deleting *btrS* on innate immune cell recruitment during infection, we enumerated neutrophils and macrophages recruited to the lungs of mice challenged with the mutant and wild-type strains^71,72^. By three days post-inoculation, lungs of wild-type infected mice showed a modest increase in numbers of neutrophils compared with naïve mice (Fig. 3D). However, mice challenged with RB50Δ*btrS* had almost three times as many neutrophils in their lungs. These numbers decreased by day 7, but still remained significantly higher than RB50-infected mice. These results indicate that BtrS is required to suppress neutrophil recruitment to the lungs.

Wild-type bacteria did not substantially increase numbers of macrophages in the lungs over control mice. However, challenge with RB50Δ*btrS* increased macrophage numbers in the lungs at day 14 post-challenge by over 60% (Fig. 3E), suggesting that BtrS is involved in the suppression of macrophage recruitment. Together, these data demonstrate that *btrS* mediates suppression of recruitment and retention of macrophages and neutrophils, potentially modulating their important roles in the generation of adaptive immunity.

### Rapid clearance of the *btrS* mutant requires TLR5

The increased neutrophil and macrophage recruitment in response to similar or lower numbers of mutant bacteria led us to consider whether BtrS modulates expression of bacterial molecules that contribute to inflammation. The increased expression of flagella genes and motility in the mutant strain and its rapid clearance suggests an immunological mechanism potentially linking these observations^21,73–77^. Flagella are known to be agonists of TLR5, raising the possibility that BtrS might mediate suppression of flagellar expression to prevent a flagellin-TLR5 response. To examine the role of TLR5 activation in the robust response to and clearance of the Δ*btrS* strain, we compared the ability of wild-type and mutant bacteria to colonize, infect and persist in TLR5-deficient mice. Our results revealed that TLR5-deficient mice did fail to rapidly clear the Δ*btrS* mutant (Fig. 3F,G), indicating that the rapid clearance of this mutant by wild-type mice is TLR5-mediated. These data provide additional evidence that the phenotype of this mutant is not simply due to the effects of BtrS on T3SS expression, but rather suggest that BtrS-mediated suppression of flagellin expression prevents a TLR5 response that would otherwise substantially augment protective immunity.

### *btrS* modulates T cell recruitment to lungs

**Figure 4:**
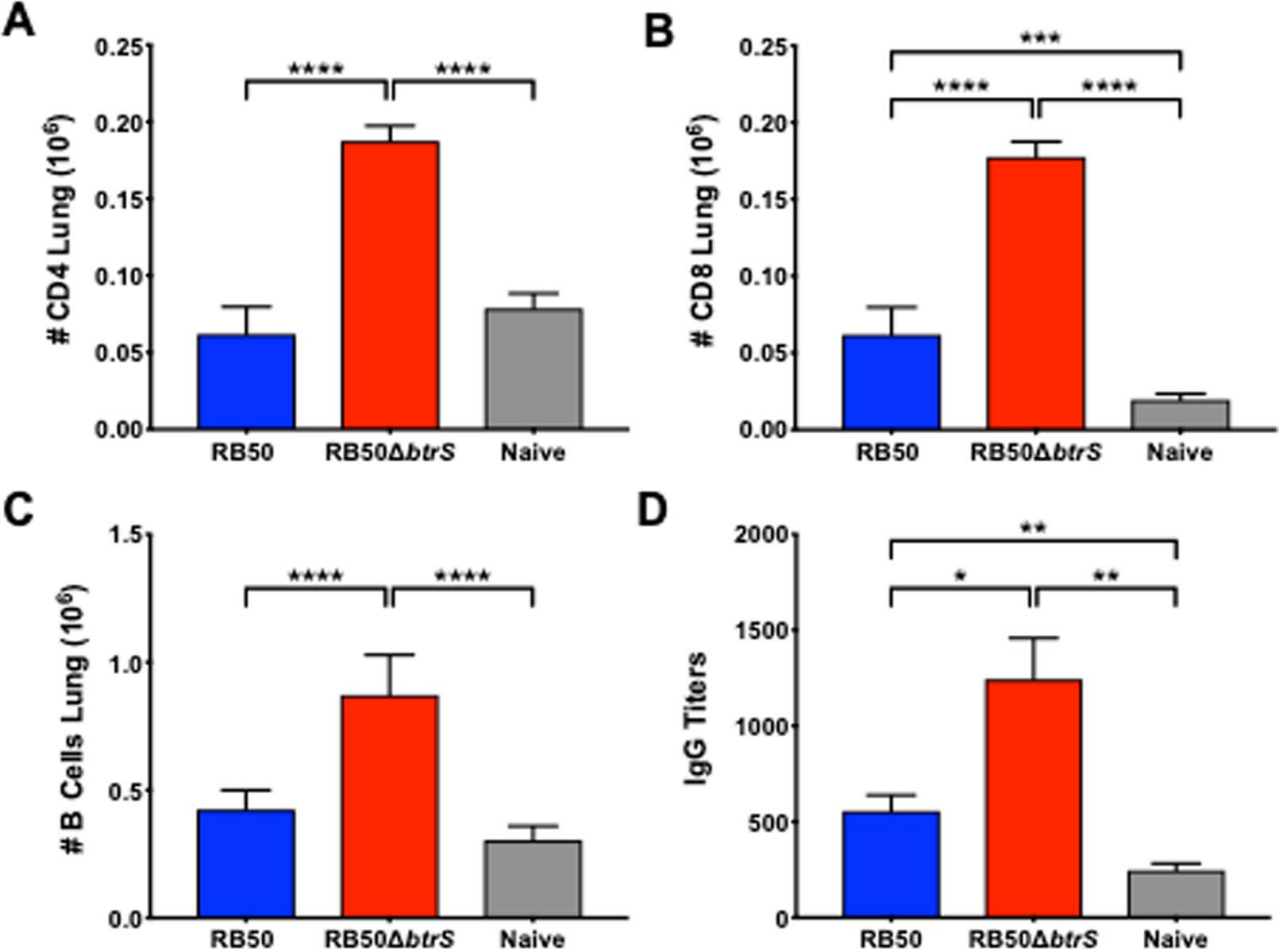
*btrS* dampens the host adaptive response. Number of CD4+ T cells (**A**), CD8+ T cells (**B**), and B cells (**C**) in the lungs of mice at day 14 post-infection with *B. bronchiseptica* RB50 (blue), RB50Δ*btrS* (red) or naïve control (grey). Anti-*B. bronchiseptica* IgG antibody titers (**D**) at day 10 post-infection (4–6 mice per group, n = 5). Statistical significance was calculated using Two-Way ANOVA. *p < 0.05, **p < 0.005, ***p < 0.0005, and ****p < 0.0001. Error bars indicate SEM, ND stands for Not Detected.

The rapid clearance of the Δ*btrS* mutant strain and the increased recruitment of innate cells in the lungs prompted a histological examination of immune cell recruitment to the lungs, which revealed both substantial lymphocyte recruitment by the mutant as well as unique distribution of those cells. Histological sections of lungs revealed the formation by day 14 p.i. of pronounced Broncho-alveolar Associated Lymphoid Tissue (BALT) in the lungs of mice challenged with the Δ*btrS* mutant but not wild type (Fig. S3C). Analysis of T cell numbers in the lungs 14 days post-inoculation via flow cytometry (Fig. S3)^71,72^ revealed that infection with wild-type RB50 did not significantly increase CD4^+^ T cell numbers in the lungs (Fig. 4A), and only modestly increased CD8^+^ T cells (Fig. 4B) compared to the non-infected control, while challenge with the Δ*btrS* mutant resulted in more than twice as many CD4^+^ and CD8^+^ T cells in the lungs. It is important to highlight that at this time point (day 14), infection with RB50Δ*btrS* induced a much greater T cell response even though the mutant was present at roughly 1/1000 the numbers of the wild-type strain (Fig. 3C). This suggests that wild-type bacteria, via mechanisms that require BtrS, can substantially block the recruitment of T cells and the formation of organized local lymphoid organoids (BALT), allowing it to grow and persist at high numbers in the lungs.

### *btrS* suppresses B cell recruitment

RB50Δ*btrS* induced macrophages to produce significantly higher levels of CCL18 and IL-6 than did wild-type bacteria (Supplementary Dataset 2 and Fig. S2F), suggesting the mutant might increase recruitment of B cells to the site of infection. Inoculation with wild-type RB50 only slightly increased B cell numbers in lungs compared with the non-infected control, whereas RB50Δ*btrS* more than doubled B cells numbers in lungs (Fig. 4C). The anti-*B. bronchiseptica* serum IgG antibody titers induced by the mutant were measurably higher as early as day 7 post-inoculation (Fig. 4D). This is a striking finding, since antibodies are generally not detectable this early after initial colonization, indicating that BtrS mediates robust B cell suppressive mechanism(s) that delay and/or reduce anti-*B. bronchiseptica* antibody titers.

### Clearance requires adaptive immunity

To investigate if rapid clearance of RB50Δ*btrS* is mediated by the T and B cells it rapidly recruits, wild-type and Δ*btrS* mutant were compared in Rag^−/−^ mice, which lack T and B cells. 21 days post inoculation both wild-type and mutant bacteria were recovered in similar numbers from all organs of the respiratory tract, indicating that the profound defects of the Δ*btrS* mutant are B and T cell dependent. To ensure that these results were not affected by the relatively large inoculum potentially overcoming the remnant immune functions in these immunodeficient animals, we performed a second challenge in which mice were intranasally inoculated with 5 μl of PBS containing 150 bacteria. 24 days later, RB50 and RB50Δ*btrS* were both recovered in similarly high numbers (Fig. S4) indicating that the mutant is not defective in any of the functions necessary for colonization, growth and spread to the lower respiratory tract, although slightly lower numbers were recovered from the nasal cavity. The observations that *btrS* is required for persistence in wild-type mice but does not affect infection and persistence in trachea and lungs of T and B cell-deficient (Rag^−/−^) mice indicates that BtrS is required for the disruption of a robust host adaptive immune response that is otherwise able to completely clear this notoriously persistent bacterium.

### The Δ*btrS* mutant induces sterilizing protective immunity to *B. bronchiseptica*

**Figure 5:**
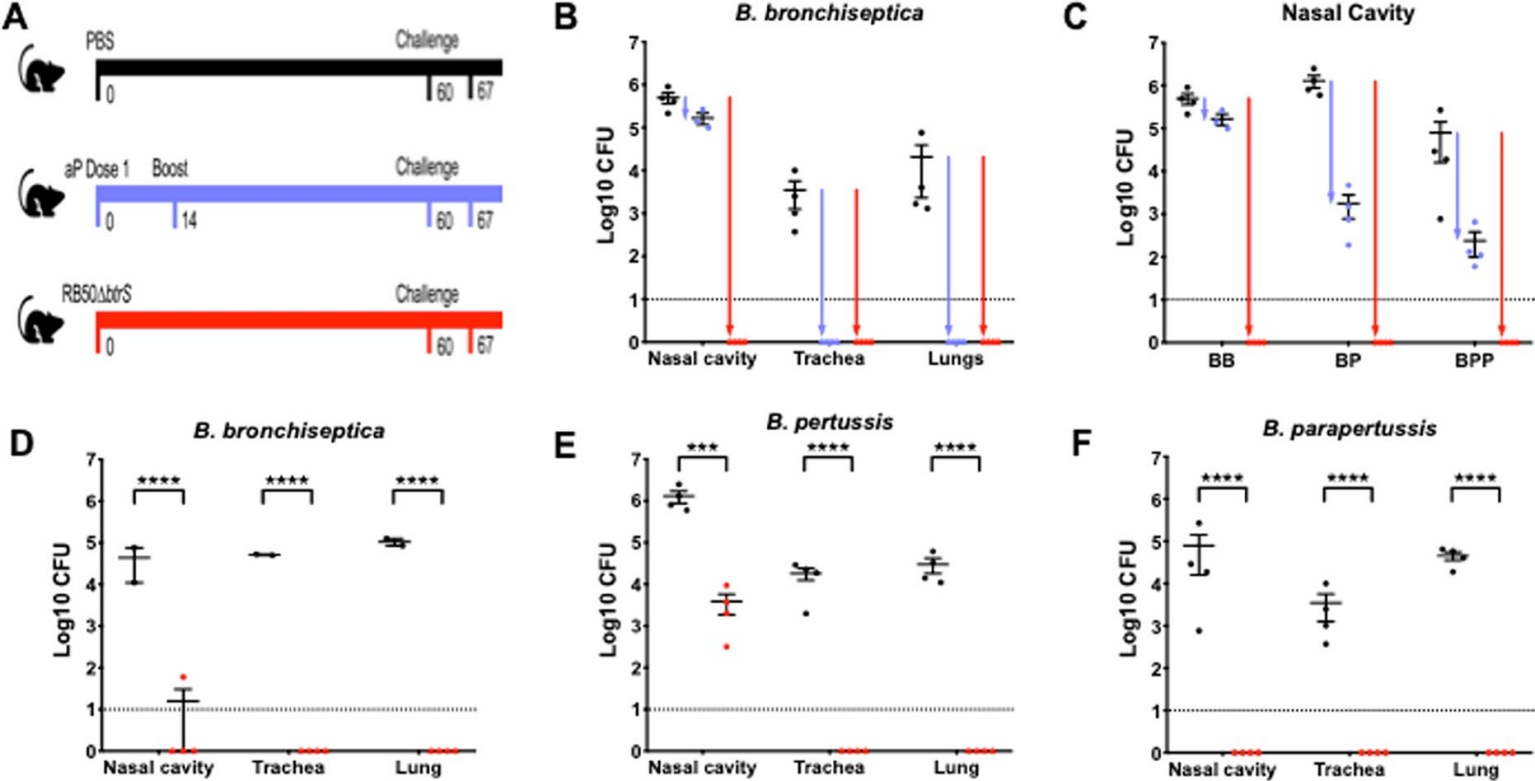
Vaccination with RB50Δ*btrS* induces sterilizing immunity to classical bordetellae. Workflow of the vaccination experiment; PBS control (black), mice vaccinated with Adacel (violet), and mice vaccinated with the RB50Δ*btrS* mutant (red). (**A**) Number of CFU recovered from the nose, trachea, and lungs of mice at day 7 post re-challenge with *B. bronchiseptica* RB50. (**B**) Number of CFU in the nasal cavity 7 days after re-challenge with *B. bronchiseptica* (BB), *B*. *pertussis (BP)*, or *B. parapertussis (BPP)*. (**C**) CFU recovered from the respiratory tract of mice vaccinated with 5 ml of PBS containing 5 CFU of RB50Δ*btrS* and re-infected with *B. bronchiseptica* (**D**), *B. pertussis* (**E**), or *B. parapertussis*. (**F**) 4–5 mice per group, n = 3. Statistical significance was calculated using Two-Way ANOVA. *p > 0.05, **p > 0.005, ***p > 0.0005, and ****p > 0.0001. The dotted line indicates the limit of detection.

The substantially increased recruitment of B and T cells leading to rapid and complete clearance of the Δ*btrS* mutant suggests that it induces robust immunity that could protect against subsequent infection. To test this, protective immunity was evaluated in three groups of mice that were 1) challenged with RB50Δ*btrS*, 2) vaccinated and boosted with the commercial acellular pertussis vaccine Adacel, or 3) administered PBS as a control. Two months later, mice were intranasally challenged with 5 × 10^5^ wild-type *B. bronchiseptica* (schematic in Fig. 5A). PBS-treated mice (control group) showed high levels of colonization across the entire respiratory tract 7 days post-challenge (Fig. 5B). Mice previously vaccinated with the acellular vaccine eliminated bacteria from the trachea and lungs, demonstrating protection against disease that is conferred by this vaccination (Fig. 5B). However, Adacel vaccination provided no significant reduction in the number of bacteria isolated from the nasal cavity. Thus, despite conferring protection of the lower respiratory tract, the acellular vaccine does not prevent colonization of the nasal cavity, allowing for transmission of the disease, consistent with clinical observations and previous results in mice and baboons^78,79^. Importantly, mice convalescent from challenge with the Δ*btrS* mutant were completely free of *B. bronchiseptica* in the lungs, trachea, and nasal cavity, indicating that this mutant confers completely protective sterilizing immunity throughout the respiratory tract (Fig. 5B). This is both qualitatively and quantitatively greater protective immunity than has been previously reported for any vaccine against *B. bronchiseptica*.

### The Δ*btrS* mutant induces sterilizing protective immunity to *B. pertussis* and *B. parapertussis*

Since the protective immunity generated by the Δ*btrS* mutant against *B. bronchiseptica* is substantially improved over that conferred by the acellular pertussis Adacel vaccine, and we have previously demonstrated variable cross-immunity between classical *Bordetella* spp.^80–83^, we tested whether immunity induced by the Δ*btrS* mutant could also protect against the closely related and antigenically similar human pathogens, *B. pertussis* and *B. parapertussis*. PBS-treated control mice challenged with *B. pertussis* or *B. parapertussis* contained high bacterial numbers in all respiratory organs at day 7 post-challenge (Figs. 5C, S5A,B). Vaccination with Adacel prior to infection resulted in reduced numbers of bacteria in the lower respiratory tract, but all three *Bordetella* species were still able to persist in the nasal cavity at day 7 post-challenge (Figs. 5C, S5A–C). These results are consistent with clinical and laboratory findings that acellular vaccination confers protection against disease, but is not completely successful at preventing colonization and transmission^79^. In contrast, mice convalescent from prior exposure to the Δ*btrS* strain were completely protected against *B. pertussis* and *B. parapertussis* colonization of the nasal cavity, trachea, and lungs at day 7 post-challenge coinciding with the peak of colonization in naïve animals (Figs. S5A–C, and 5C), demonstrating fully sterilizing immunity against all three species.

To determine if a very low dose of the Δ*btrS* mutant is sufficient to provide protection against the classical *Bordetella* species, mice were challenged with 5 CFU of the mutant and 60 days later challenged with *B. bronchiseptica*, *B. pertussis*, or *B. parapertussis*. While naive control animals were colonized to high levels, prior exposure to 5 CFU of the Δ*btrS* mutant resulted in nearly complete protection against colonization by *B. bronchiseptica* (Fig. 5D) and *B. parapertussis* (Fig. 5F). Prior exposure to 5CFU of of the Δ*btrS* mutant resulted in complete protection against *B. pertussis* colonization of the lower respiratory tract and greater than 99% reduction in numbers in the nasal cavity.

In all cases, exposure to even very low numbers of the Δ*btrS* mutant effectively protected against all three classical *Bordetella* species.

## Discussion

During the course of infection, bacteria are confronted with an ever-changing assortment of antimicrobials in a variety of challenging host micro-environments with varying levels of inflammation and adaptive immune functions^10,28,29,84–93^. The host immune response involves a complex series of signals that recruit and activate a succession of immune cells to confront the bacterial threat. Yet successful pathogens, such as *Bordetella* spp., rapidly respond to, suppress and evade host immunity to persist and mediate their own transmission to new hosts, and can even lay the groundwork for future reinfection of the same host^94–96^. Our results indicate that by deleting a single bacterial sigma factor, *btrS*, we were able to interrupt the ability of *B. bronchiseptica* to suppress effective host adaptive immunity, indicating that this sigma factor is a key regulator of immunomodulatory functions including T3SS, flagella, and membrane proteins. BtrS appears to mediate an increase in inflammasome activation and macrophage cell death, conditions that would be expected to cause a deficient adaptive immune response, as observed here and as previously reported for other bacterial species^63–65^. The BtrS-mediated decrease in chemokine production by macrophages and immune cell recruitment may explain the reduction in B and T cell recruitment that delays clearance from the lower respiratory tract and allows long-term persistence in the upper respiratory tract. The robust immune response elicited by challenge with this mutant led to protection against further challenge with any of the three classical bordetellae. Importantly, this protection was even better than that conferred by convalescent immunity with the wild-type strain, highlighting that these immunomodulatory abilities can also suppress protective memory^97^. Even 5 CFU of the mutant is enough to robustly protect against further challenge with a half-million CFU of *B. bronchiseptica*, *B. pertussis*, or *B. parapertussis*. Together, these data demonstrate that the sigma factor BtrS is involved in regulation of numerous genes and that these collectively mediate profound immunomodulation.

Although we previously showed that TLR4-deficient mice are highly susceptible to *B. bronchiseptica*^98–101^, TLR5-deficient mice were not highly susceptible to *B. bronchiseptica*, indicating that its detection of Microbe-Associated Molecular Patterns (MAMPs) such as flagellin are not crucial to the normal immune response to the wild-type strain. Combined with transcriptional data demonstrating that *btrS* is required to suppress flagellin expression, these data lead to a compelling model: BtrS mediates the suppression of the TLR5 agonist flagella to prevent the generation of more effective protective immune response. In 1995, Akerley *et al*. demonstrated that Bvg is important not only for activation of virulence factors, but also for the suppression of other factors^21^. In that study, ectopically expressing flagella during infection led to a severe defect in persistence in rats and rabbits. At the time, it was known that flagella can be highly immunogenic, but its effects on TLR5 were yet to be discovered. Our results revealed that the mutant lacking BtrS fails to suppress flagella expression and induces robust protective immunity that is TLR5-dependent, suggesting that the careful regulation of potential immunostimulants is critical for successful infection and persistence. Our results suggest that TLR5 contributes to the generation of much more robust protection that can be cross-protective against other *Bordetella* species an area of investigation that could lead to the development of improved vaccines against these and other pathogens^75–77^. These results are consistent with the observations that addition of TLR5 agonists have increased the performance of vaccines against malaria, bubonic plague, flu, *Enterococcus* spp., and *Francisella tularensis*^102–109^. Our results suggest that TLR5 agonists could significantly increase the performance of the current acellular pertussis vaccine.

Overall, the evidence that BtrS is a regulator of immunomodulatory functions provides a novel perspective on *Bordetella*-host interactions. The SigE family of sigma factors is conserved among a wide range of bacterial species, but despite intriguing evidence that implicates them in host-pathogen interactions^41,42,52,110,111^, their *in vitro* roles in stress response are better understood than their roles *in vivo*. Efficient natural-host infection and mouse immunological tools available in *Bordetella* spp. experimental infection systems allow such interactions to be probed in detail. Our results reveal that regulators such as sigma factors can respond to host signals and play important roles in manipulation of host immune responses. Moreover, their roles appear to be significantly more sophisticated than the linear control of one or two virulence factors and may not be accurately simulated *in vitro*. Exquisite *in vivo* regulation of immunomodulatory mechanisms, such as secretion systems, flagella, toxins, modulins, adjuvants and antigens, is likely to be an important facet of efficient infection and pathogenesis of other well-adapted pathogens.

Immunity observed in convalescent animals is commonly believed to be the “gold standard” against which vaccines are compared. However, this concept is in conflict with substantial evidence that many well-adapted pathogens modulate immunity and, as in the case of *B. pertussis*, do not provide lifelong immunity. The results of this work suggests that *Bordetella* spp. harbor mechanisms that are finely tuned to modulate the host immune response, enabling them to increase persistence while modulating inflammation and immunity to enhance their opportunity to transmit to another host. The rapid and robust innate and adaptive immunity that resulted in early clearance of the *btrS* mutant is measurably increased over that induced by the wild-type strain, raising questions about whether natural immunity to infection with this and other *Bordetella* species should be considered the standard against which vaccines are judged. The ability of the *btrS* mutant to confer protective immunity against further encounters with this and related *Bordetella* species demonstrates that immunity induced by infection is substantially suppressed and should not be considered the “gold standard” for optimal immunity. Our vision of what is possible should set our sites much higher, as we have now demonstrated that significantly better protection is possible.

## Materials and Methods

### Bacterial strains and culture conditions

*Bordetella* spp. were grown on plates of Difco Bordet-Gengou agar (BD, cat. 248200) supplemented with 10% sheep defibrinated blood and 20 μg/mL working concentration of streptomycin^28,112^. Strains used in this study are the same strains of *B. bronchiseptica*, *B. pertussis* and *B. parapertussis* used in our previously published manuscript^28^. Knock out mutants were generated as previously described^5,22,112–115^.

### Complemented strain generation

PCR was used to amplify *btrS* as well as to linearize the plasmid pYS003 (a pBBR1-mcs5 derivative). pBBR-*fhaB*-GFP was derived from pLC007(pBBR-gfp) by swapping the lac promoter of GFP with *Bordetella bronchiseptica* fhaB promoter region. The parental vector pLC007 was linearized by PCR with primers YS031 & YS032 to delete the lac promoter regions (YS031_PIPEve_pLC009_F CACATTAATTGCGTTGCGCTCACTGC//YS032_PIPEve_pLC009_R ATGAGTAAAGGAGAAGAACTTTTCACTGGA). The *fhaB* promoter region was amplified by PCR with YS037 & YS038 (YS037_PIPEin_fhaB_F 5′-3′ GCAACGCAATTAATGTGcggtttcgccgatgacttcgaatc//YS038_PIPEin_fhaB_R 5′-3′ CTTCTCCTTTACTCATattccgaccagcgaagtgaagtaatc). In both cases, PCR was performed without the final extension step in order to leave single-strand DNA fragments on both ends of the products (PIPE Cloning PCR^116^). The plasmid was digested with Dpn1 (BioLabs, cat. R0176S) and reamplified. The two products were then purified again and ligated. The plasmid was extracted and electroporated into competent knockout mutant bacteria (RB50Δ*btrS*) at 2500 V. The presence of the *btrS* insert in RB50Δ*btrS* and in DH5α was verified via PCR.

### Macrophages assays

#### Internalization and intracellular survival assay

Intracellular survival in RAW264.7 and THP-1 cell lines was assessed following the traditional gentamicin protection assay method^117^. In brief, macrophages were challenged at MOI of 100, followed by centrifugation at 300 g for 5 minutes. After 1 hour, after which the media was replaced by media containing 300 μg/ml of gentamicin. Antibiotics were maintained for the whole length of the experiment. For all consecutive experiments, we also followed this approach (adding gentamicin after the first hour of incubation). To measure internalization, cells were washed twice with PBS followed by 5 minutes incubation in ice with 1% Triton (Invitrogen, cat. HFH10). The recovered bacteria were plated on BG/strep plates and enumerated 48 hours later. For the intracellular survival assays, we followed the same procedure at all different time points specified on the graphs. All the experiments were performed in triplicate; see details of the numbers of replicates on each figure.

#### Transmission electron microscopy

Samples were fixed at different times (15 minutes and 4 hours post-challenge) with 2% glutaraldehyde.

#### Cytokine production

To asses IL-1β, TNF-α and IL-6 expression, the supernatant of infected cells was utilized to quantify levels of cytokines via commercially available ELISA kits (DuoSet ELISA system, R&D Systems)^118^. Inflammasome activity was evaluated following the protocol of the Caspase-Glo 1 Inflammasome Assay Kit (Promega, cat. G9951).

#### Cytotoxicity assay

Briefly, RAW murine macrophages RAW264.7 were cultured in RPMI medium 1640 (Gibco, Life Technologies) supplemented with 10% Fetal Bovine Serum (FBS) (Gibco, Life Technologies) and 100 U/ml Penicillin-Streptomycin (Gibco, Life Technologies) to 95% confluency. Then macrophages were washed twice with RPMI supplemented with 2% FBS and lacking antibiotics to allow bacterial infection. Multiplicity of infection was 100 (MOI 1:100). After the bacterial addition samples were centrifuged at 300 g for 10 min and incubated for 15 minutes or 4 h at 37 °C in a 5% CO_2_ atmosphere. The cytotoxicity was measured as the release of LDH using the CytoTox 96 Kit (Promega) following the manufacturer’s protocol. As controls, we performed the same assay on media containing bacteria at the same concentration, lacking macrophages, confirming that no-cytotoxicity is detected.

#### RNA sequencing

RNA was extracted following manufacturer’s recommendations of the RNAeasy Kit (Qiagen, cat. 74104) as previously detailed^28,62^.

#### Bioinformatics

RNA was sequenced on an illumina Hiseq platform by Mr. DNA Lab, Shallowater, TX. For the analysis of the response of *B. bronchiseptica* to medium and serum, a total of 15 to 18 million 2 × 150 bp reads were obtained for each sample. For the analysis of the macrophage transcriptome in response to *B. bronchiseptica* infection, approximately 500 million 2 × 150 bp reads were obtained for each sample. Quality control, trimming, and mapping were performed using the CLC Genomics v.11 analysis suite. Samples were mapped either to the transcriptome of *B. bronchiseptica* or *Mus musculus* and changes in gene expression analyzed by performing an empirical analysis of DGE^119^. Changes in gene expression were considered significant if the false discovery rate was <0.05. Additional data analysis included gene set enrichment analysis (GSEA^120^) and string analysis^121^. Datasets were deposited at SRA (Bioproject numbers PRJNA559660).

#### Enzyme-linked immunosorbent assays

96-well microtiter plates (Costar) were coated with heat-killed *B. bronchiseptica* wild-type or mutant as previously reported^67^. SureBlue (SeraCare, cat. 5120-0076) was added to start the reaction, which was terminated with HCl after three minutes. The plates were read at an OD of 450 nm. The titer was determined to be the reciprocal of the lowest dilution in which an OD of 0.1 was obtained.

### Animal experiments

Wild-type C57BL/6J and Rag−/− (B6.129S7 Rag1tm1Mom/J) mice were obtained from Jackson Laboratories, Bar Harbor, ME or our breeding colony (established from Jackson laboratories mice). Mice were bred and maintained at Paul D. Coverdell Center for Biomedical and Health Sciences, University of Georgia, GA, (AUP: A2016 02-010-Y2-A3)^28,122^. All experiments were carried out in accordance with all institutional guidelines (*Bordetella* Host Interactions AUP: A2016 02-010-Y2-A6). Nasal cavity, trachea, lungs, spleen and blood were collected post-mortem in 1 mL of cold PBS. When tissues were used to enumerate colonies, collection was performed in beaded tubes, and when organs were used for immunology, they were collected in 15 mL falcon tubes containing sterile PBS. All results were graphed in GraphPrism v8 and statistical significance was calculated using two-way ANOVA. A power analysis was used (G-Power 3) to compare the immunological response and colonization and we utilized at least 6 mice per group (and experiments were performed in triplicate) at alpha 0,05 and power of 80%.

#### Flow cytometry

Spleen and lungs were processed and stained as previously described^71,72^. Numbers of live cells were enumerated with Countess II (Thermo Fisher) with trypan blue stain. Two million live cells were seeded in each well for staining. Antibody panels are shown in Supplementary Table S1. The acquisition of the data was performed using BD-LSR II (Becton Dickinson) and analysis was performed with FlowJo 10.0 following standard gating strategy. Statistical significance was calculated using two-way ANOVA in GraphPrism.

#### Ethics statement

This study was carried out in strict accordance with the recommendations in the Guide for the Care and Use of Laboratory Animals of the National Institutes of Health. The protocol was approved by the Institutional Animal Care and Use Committee at the University of Georgia, Athens (A2016 02-010-Y2-A3 Bordetella-Host Interactions and A2016 07-006-Y2-A5 Breeding protocol). All animals were anesthetized using 5% isoflurane and euthanized using carbon dioxide inhalation followed by cervical dislocation to minimize animal suffering. Animals were handled following institutional guidelines, in keeping with full accreditation from the Association for Assessment and Accreditation of Laboratory Animal Care International.

## Supplementary information

Supplementary Information

Supplementary Information 2

Supplementary  Information 3
