# Supplementary material for: Enhancement of immune response against Bordetella spp. by disrupting immunomodulation
Source: Sci Rep. 2019 Dec 30;9:20261. doi: 10.1038/s41598-019-56652-z (PMC6937331; doi:10.1038/s41598-019-56652-z)
Supplement: Supplementary file 1 — Supplementary Information [file 41598_2019_56652_MOESM1_ESM.pdf]

Title: Enhancement of immune response against *Bordetella* spp. by disrupting immunomodulation

Monica C. Gestal[1]*, Laura K. Howard[1], Kalyan Dewan[1], Hannah M. Johnson[1], Mariette Barbier[2,3], Clare Bryant[4], Illiassou Hamidou Soumana[1], Israel Rivera[1], Bodo Linz[1], Uriel Blas-Machado[5], and Eric T. Harvill[1]*.

[1]Department of Infectious Diseases, College of Veterinary Medicine, University of Georgia, Athens, Georgia, United States of America.

[2]Department of Microbiology, Immunology, and Cell Biology, West Virginia University, 9 Morgantown, WV, United States of America.

[3]Vaccine Development Center at West Virginia University Health Sciences Center, Morgantown, West Virginia, USA.

[4]Department of Veterinary Medicine, University of Cambridge, Cambridge CB3 0ES, United Kingdom.

[5]Department of Pathology, Athens Veterinary Diagnostic Laboratory, University of Georgia, Athens, Georgia, United States of America.

Corresponding authors:

Monica Cartelle Gestal: mcarges@gmail.com // mcgestal@uga.edu

Eric T. Harvill: Harvill@uga.edu

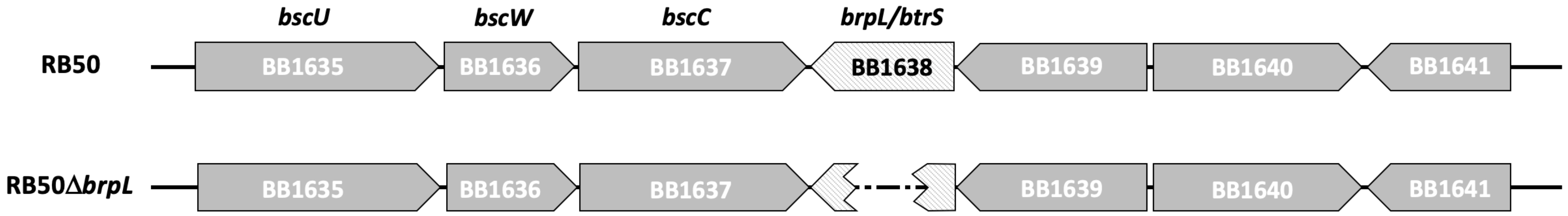

Figure S1: Genetic context for RB50Δ*btrS* mutant strain. Diagram showing the clean, in-frame deletion of the *brpL*/*btrS* gene (Locs_tag BB1638) in the RB50 *B. bronchiseptica* chromosome.

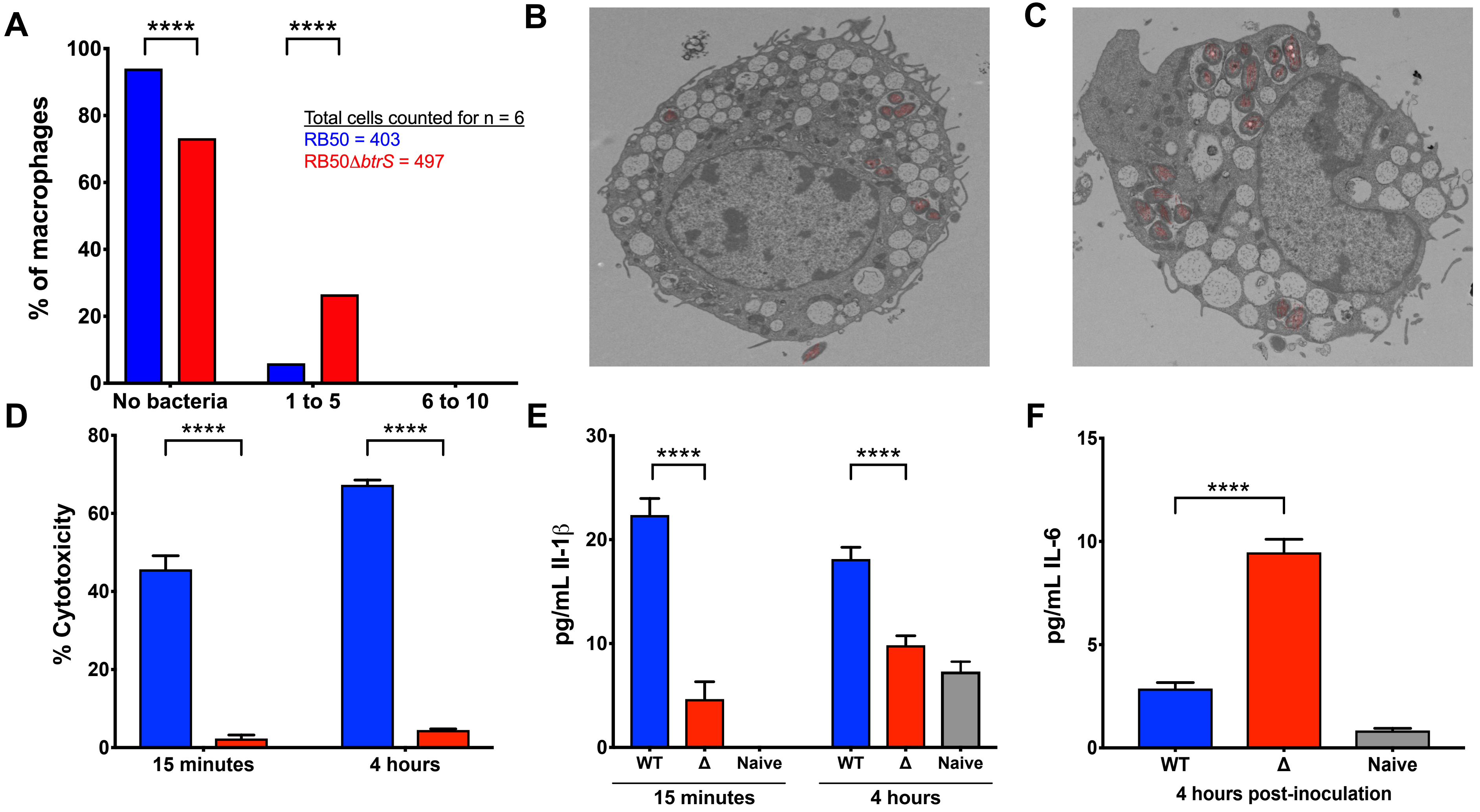

Figure S2: Macrophages at a confluency of 97-99% were challenged at an MOI of 100, n=6. (**A**) Percentage of macrophages containing the indicated number of bacteria at 15 minutes post-exposure. RB50 (blue) and RB50Δ*btrS* (red) were enumerated using Transmission Electron Microscopy (TEM). TEM of RAW macrophages challenged for 4 hours with RB50 (**B**) (bacteria are shown in red) or with the mutant (**C**). (**D**) Cytotoxicity of RB50 and RB50Δ*btrS* at 15 min and 4 hours post-challenge. (**E**) IL-1β secreted measured at 15 min and 4 hours post-challenged of macrophages challenged with wild-type or mutant *B. bronchiseptica*. (**F**) IL-6 secreted at 4 hours post-challenge with RB50 or RB50Δ*btrS*. Statistical significance was calculated using Two-Way ANOVA. * $p<0.05$, ** $p<0.005$, *** $p<0.0005$, and **** $p<0.0001$. Error bars indicate SEM.

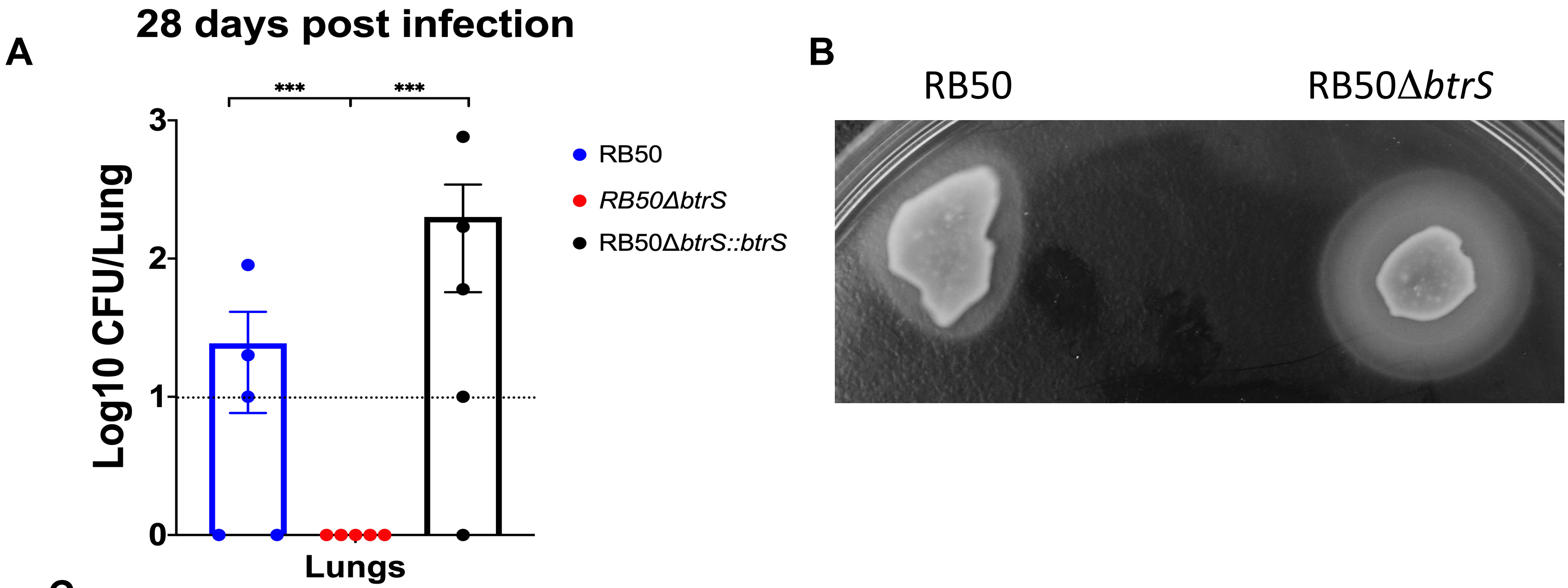

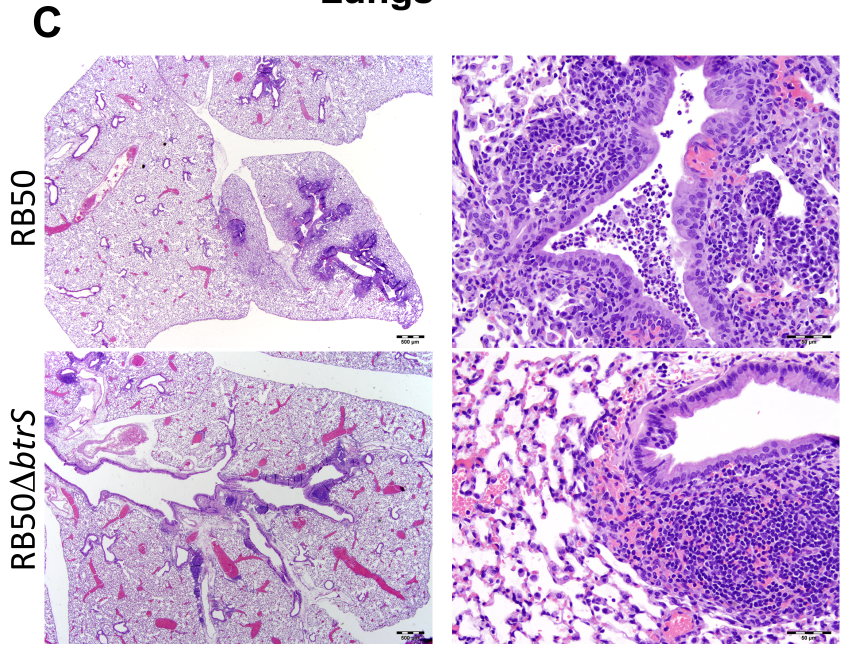

Figure S3: Phenotypes that required *btrS in vivo* and *in vitro.* (**A**) Mice were intranasally challenged with RB50 (blue), RB50Δ*btrS* (red), or RB50Δ*btrS::btrS* (black). 28 days post-inoculation organs were harvest and bacterial load in the lungs was enumerated. (**B**) Motility assay was performed in 0.4% LB agar inoculated with 10μl of a liquid culture of RB50 or RB50Δ*btrS* at an OD of 0.7. The plates were read at 24 and 48 hours post-inoculation (n=4) (**B**) Mice were intranasally challenged with RB50 or RB50Δ*btrS* and pathological studies (H&E) were performed. In the left is the zoom out and the right is the zoom in image of RB50 (top) and RB50Δ*btrS* (bottom).

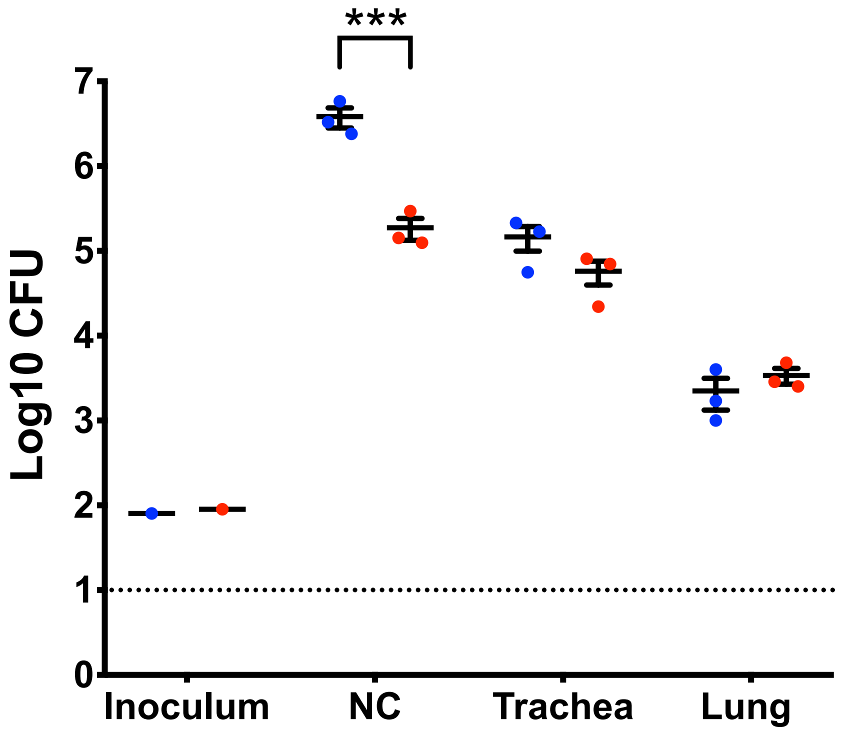

Figure S4: Bacterial numbers isolated from nasal cavity, trachea, and lungs of Rag$^{-/-}$ mice.· Groups of 4 mice were challenged with a low dose (150 CFU) in a low volume (5mL) of wild-type (blue) or mutant (red) *B. bronchiseptica* at 24 days post-inoculation. Bacterial load from the respiratory tract organs was enumerated (n=2). Statistical significance was calculated using Two-Way ANOVA. *** $p<0.0005$. Error bars indicate SEM.

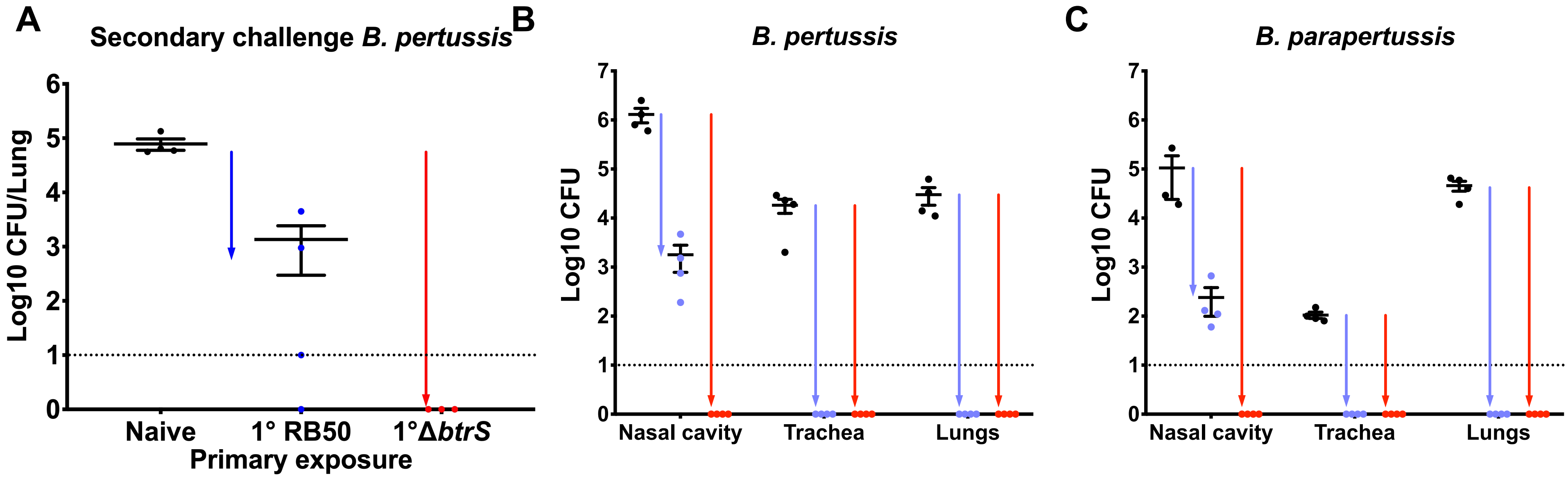

Figure S5: Efficiency of RB50Δ*btrS* as a vaccine against *B. pertussis* and *B. parapertussis*. (**A**) Mice were challenged with PBS (black), RB50 (blue), or RB50Δ*btrS* (red). 3 months post-exposure, mice were inoculated with *B. pertussis* and sacrificed at day 7 (n=4). Bacterial load in the lungs was enumerated. (**B-C**) Mice were vaccinated with PBS (black), Adacel acellular vaccine (violet), or RB50Δ*btrS* (red). 60 days later, mice were re-challenged with (**B**) *B. pertussis* or (**C**) *B. parapertussis* and euthanized at day 7 (n=4).

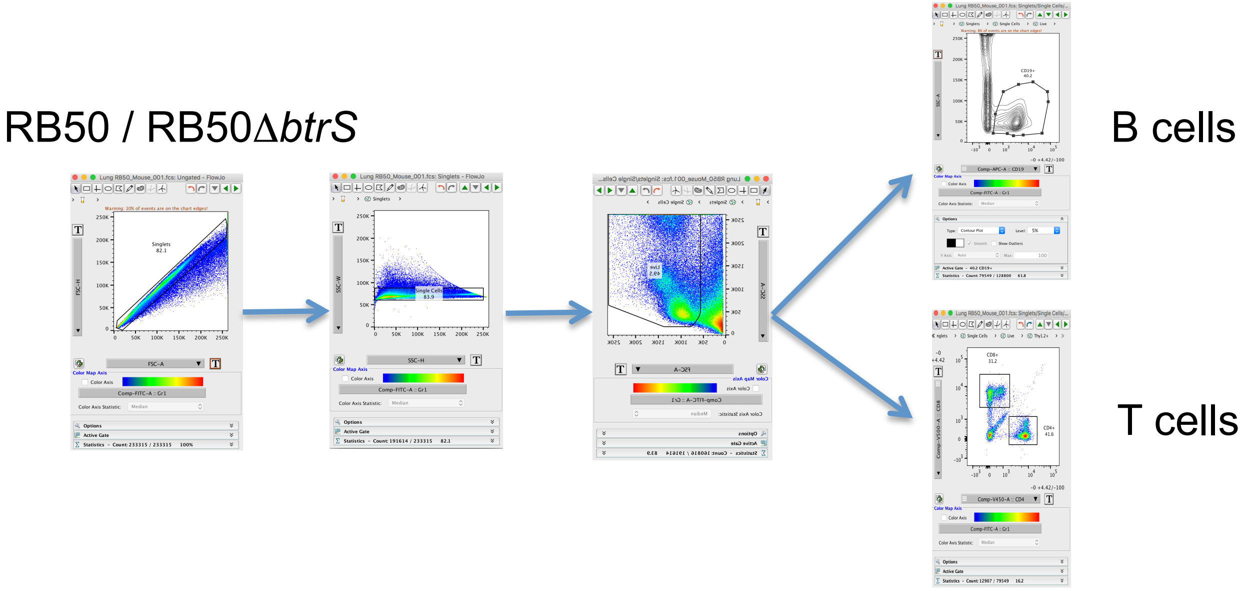

Figure S6: Gating strategy utilized for flow cytometry. Strategies adapted from standard gating procedures *(52)* (n=6 per experiment of 4).

Table S1.

Antibodies used in this study are detailed on the table below.

Laser	BP filter	Fluorochromes	Cytokine	Vendor	Reference
488 Laser B	525/50	FitC	GR1	Tonbo	35-5931
532 Laser E	575/20	PE	CD11b	Tonbo	50-0112
488 Laser A	695/40	PerCP	F4/80	Tonbo	65-4801
640 Laser C	660/20	APC	CD19	Tonbo	20-0193
640 Laser A	780/60	APC-Cy7	CD90.2	BD	561641
405 Laser C	450/50	V450	CD4	Tonbo	75-0041
404 Laser B	525/50	V500	CD8	BD	563068
